# Single-cell heterogeneity and dynamic evolution of Ph-like acute lymphoblastic leukemia patient with novel *TPR-PDGFRB* fusion gene

**DOI:** 10.1186/s40164-023-00380-8

**Published:** 2023-02-17

**Authors:** Xuehong Zhang, Zhijie Hou, Dan Huang, Furong Wang, Beibei Gao, Chengtao Zhang, Dong Zhou, Jiacheng Lou, Haina Wang, Yuan Gao, Zhijie Kang, Ying Lu, Quentin Liu, Jinsong Yan

**Affiliations:** 1 Department of Hematology, Liaoning Medical Center for Hematopoietic Stem Cell Transplantation, Dalian Key Laboratory of Hematology, Liaoning Key Laboratory of Hematopoietic Stem Cell Transplantation and Translational Medicine, Diamond Bay Institute of Hematology, The Second Hospital of Dalian Medical University, Dalian, China; 2 Institute of Cancer Stem Cell, Dalian Medical University, Dalian, China; 3 Department of Neurosurgery, the Second Hospital of Dalian Medical University, Dalian, China; 4 Institute of Dermatology, Xinhua Hospital, Shanghai Jiao Tong University School of Medicine, Shanghai, China; 5 State Key Laboratory of Oncology in South China, Sun Yat-sen University Cancer Center, Guangzhou, China

**Keywords:** Philadelphia chromosome-like acute lymphoblastic leukemia (Ph-like ALL), Fusion gene, Single-cell RNA sequencing (scRNA-seq), Cellular heterogeneity, Immunotherapy

## Abstract

Philadelphia chromosome-like acute lymphoblastic leukemia (Ph-like ALL) is a refractory and recurrent subtype of B-cell ALL enriched with kinase-activating rearrangements. Incomplete understanding of the heterogeneity within the tumor cells presents a major challenge for the diagnosis and therapy of Ph-like ALL. Here, we exhibited a comprehensive cell atlas of one Ph-like ALL patient with a novel *TPR*-*PDGFRB* fusion gene at diagnosis and relapse by using single-cell RNA sequencing (scRNA-seq). Twelve heterogeneous B-cell clusters, four with strong *MKI67* expression indicating highly proliferating B cells, were identified. A relapse-enriched B-cell subset associated with poor prognosis was discovered, implicating the transcriptomic evolution during disease progression. Integrative single-cell analysis was performed on Ph-like ALL and Ph^+^ ALL patients, and revealed Ph-like specific B-cell subpopulations and shared malignant B cells characterized by the ectopic expression of the inhibitory receptor *CLEC2D*. Collectively, scRNA-seq of Ph-like ALL with a novel *TPR-PDGFRB* fusion gene provides valuable insights into the underlying heterogeneity associated with disease progression and offers useful information for the development of immunotherapeutic techniques in the future.

**Supplementary Information:**

The online version contains supplementary material available at 10.1186/s40164-023-00380-8.

**To The Editor**,

Philadelphia chromosome-like acute lymphoblastic leukemia (Ph-like ALL) is an aggressive neoplasm of B lymphoblasts, with a gene expression profile similar to that of Ph^+^ ALL but lacking the *BCR-ABL1* translocation, and 5-year overall survival does not exceed 20% [1, 2]*.* It has been reported that drug resistance and subsequent relapse induced by heterogeneous blasts remain the leading causes of death among Ph-like patients [3]. Single-cell RNA sequencing (scRNA-seq) for B-cell ALL (B-ALL) revealed the extensive remodeling of the immune microenvironment during the progression [4], pre-existing CD19 negative subclones [5], and the presence of an exhausted T cell subset with remarkable heterogeneity [6]. However, cellular heterogeneity and dynamics of Ph-like ALL at single-cell resolution remains unknown.

Previous study has been reported that patients with *PDGFRB* rearrangements account for ~ 8% of the Ph-like cases [7]. Our Ph-like ALL patient was a 55-year-old woman who harbored a novel *TPR-PDGFRB* in-frame fusion gene which consists of exons 1–46 of the *TPR* with exons 11–23 of the *PDGFRB* (Fig. 1A, and Additional file 1: Table S1, S2). Initially, this patient was diagnosed as ALL common-B based on morphology and immunophenotyping, and conventional chemotherapy regimen was started (Additional file 1: Table S3 and Fig. S1). However, she developed rapid relapse twice following short-lived remission (Fig. 1B). Previous studies have reported that *PDGFRB* fusion patients with Ph-like ALL were refractory to conventional therapy but amenable to tyrosine kinase inhibitor (TKI) [7]. Single agent of imatinib was unable to reduce the lymphoblasts within 2 weeks, and then further she developed into central nervous system leukemia (CNS-L) with 96.5% lymphoblasts (Fig. 1B). Due to the enhanced inhibitory of tyrosine kinase activity and the infiltration ability across the blood brain barrier, one course of dasatinib combined with DOCPL regimen was administered (Fig. 1B, Additional file 1: Table S3). Then, complete remission (CR) was achieved and maintained for 4 months (Fig. 1B). Ponatinib was given because of relapse and unfit for chemotherapy, unfortunately, she achieved no response and finally died after 14 months from initial diagnosis (Fig. 1B).

**Fig. 1:**
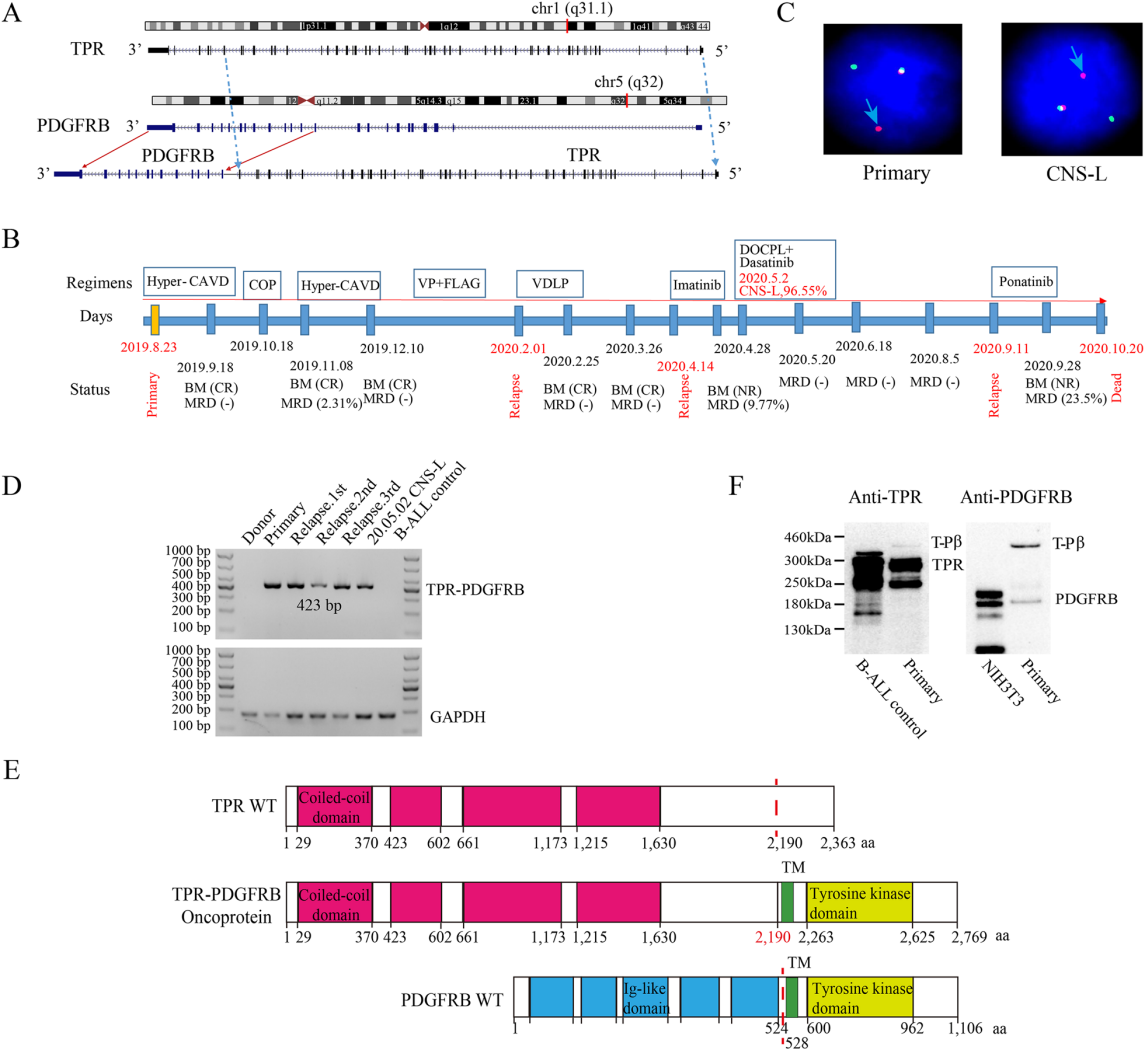
A novel *TPR-PDGFRB* fusion gene was identified in one patient with Ph-like ALL. **A** Graphical representation of the organization process for the *TPR-PDGFRB* fusion at the chromosome level. **B** Diagram of the whole treatment process of the *TPR-PDGFRB* positive patient. NR, not remission; PR: partial remission; CR: complete remission; MRD: minimal residual disease. **C** Interphase FISH analysis with *PDGFRB* break-apart probe showing a split *PDGFRB* signal pattern in primary and CNS-L blasts. The arrow indicates a break-apart signal in the *PDGFRB* gene. **D** RT-PCR was performed across the *TPR-PDGFRB* fusion gene breakpoint, and a 423-bp product from the forward primer (5′-AGTCTGTAGGACGTGGCCTT-3′) located in the exon 44 of *TPR* gene and reverse primer (5′-TGGGGTCCACGTAGATGTACTC-3′) located in the exon 12 of *PDGFRB* gene was amplified. **E** Schematic diagrams of the *TPR*, *PDGFRB*, and *TPR-PDGFRB* fusion proteins. The breakpoint is indicated by a red dashed line. TM: transmembrane domain. **F**, NIH 3T3 cells, *TPR-PDGFRB* fusion-negative B-ALL cells, and primary blasts were immunoblotted for *TPR* (Abcam #ab70610) and *PDGFRB* (CST, #3169)

Meanwhile, we evaluated the expression similarity between this patient and Ph^+^ ALL patients based on two publicly available datasets [8, 9] (Additional file 1: Additional file methods). The patient exhibited higher correlation coefficients with Ph^+^ ALLs than with other ALL subtypes (Additional file 1: Fig. S2A), and blended into the Ph^+^ cluster under the hierarchical clustering analysis on the basis of the Ph-like ALL signature (Additional file 1: Fig. S2B), indicating the Ph-like expression feature. Fluorescence *in-situ* hybridization (FISH) confirmed the *PDGFRB* break-apart (Fig. 1C), reverse transcription-polymerase chain reaction (RT-PCR) and Sanger sequencing validated the junction sequences of the *TPR-PDGFRB* fusion transcript (Fig. 1D, Additional file 1: Fig. S2C). The presumed *TPR-PDGFRB* protein retained the transmembrane and tyrosine kinase domains from *PDGFRB* (Fig. 1E), and immunoblotting confirmed the presence of chimeric protein *TPR-PDGFRB* (Fig. 1F). Furthermore, we observed the reduced and elevated frequency of *TPR-PDGFRB* fusion transcripts along with disease remission and recurrence (Additional file 1: Fig. S2D-G), suggesting the potential as a marker for evaluating the minimal residue disease (MRD) of this patient.

To decipher the cell heterogeneity and dynamics of Ph-like ALL, scRNA-seq was performed on 10,273 cells taken from the bone marrow specimens at diagnosis and first relapse (Fig. 2A, Additional file 1: Fig. S3A). Seventeen cell clusters were respectively labeled as B cells, megakaryocytes-erythroid progenitors (MEPs), T cells, natural killer (NK) cells, classical and non-classical monocytes (Fig. 2B, C, Additional file 1: Fig. S3B-F). Further, twelve B cell clusters were classified into six subsets involved in *CD34*^+^ ProB cells, *CD20* (*MS4A1*) high and *IGLL1* low PreB cells, *CD20* low and *IGLL1* high PreB cells, *IGKC* low and *IGLL1* high PreB cells, cycling PreB cells (*MIK67* high expression), and memory B cells (Fig. 2D, E, Additional file 1: Fig. S3G). Pseudotime inference revealed the distinct bifurcated architecture of cell trajectory, which appearing to start principally from the *CD34*^+^ ProB and cycling PreB cells, then moved toward two *IGLL1* high PreB cell subsets, following to *CD20* high or memory B cell subsets, implying a divergence in the transcriptional state (Fig. 2F).

**Fig. 2:**
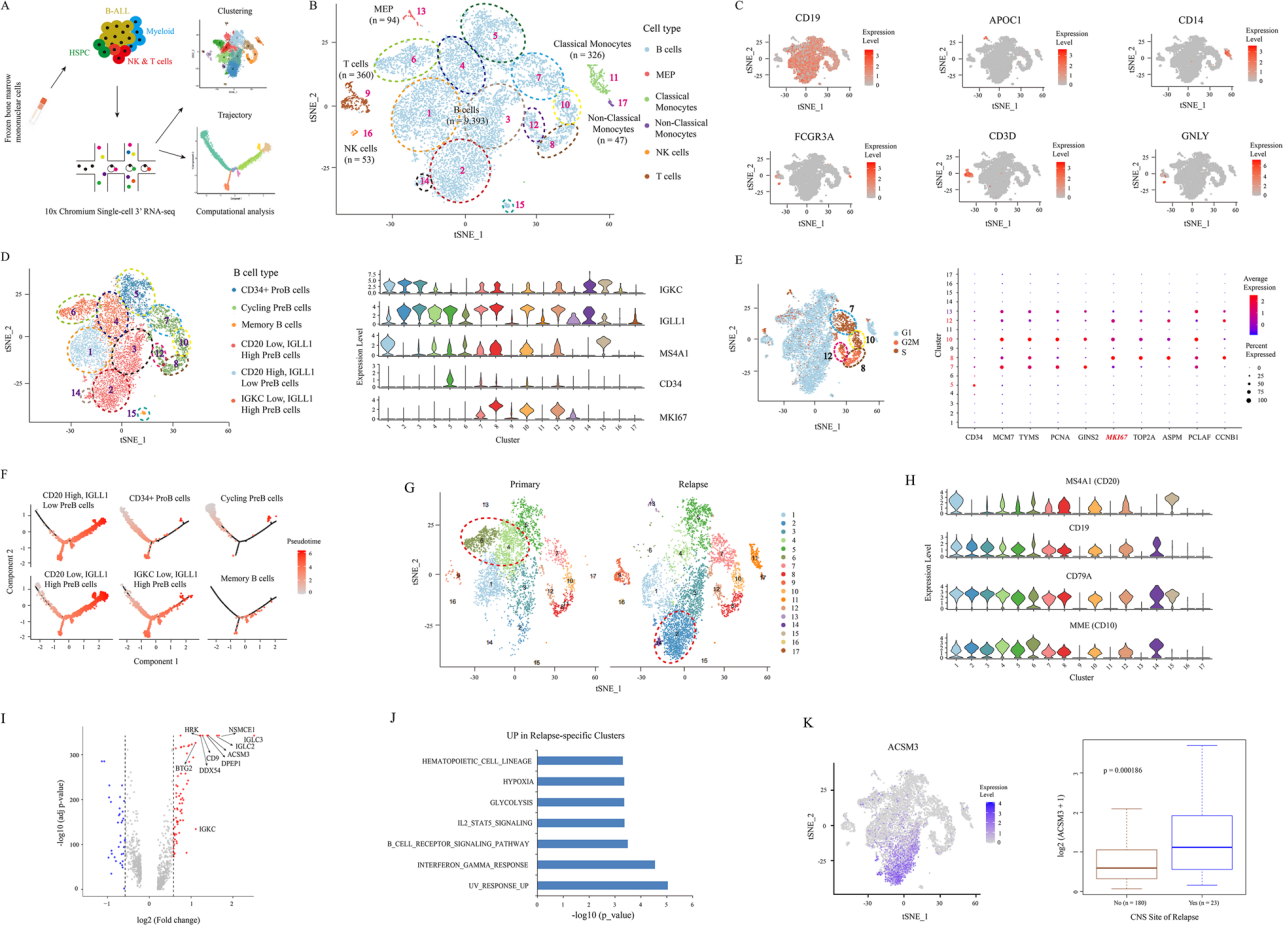
scRNA-seq reveals the transcriptional heterogeneity and dynamic evolution of patient with Ph-like ALL. **A** Schematic representation of the isolation, sequencing, and analysis of the single cells. **B** Six broad immune cell types assigned to all profiled single cells via cell type identification analysis. **C** Expression levels of lineage-specific genes overlaid on the tSNE representation. **D** Marker-based cell type identification analysis defined six B-cell subtypes. **E** tSNE plots showing the three cell cycle phases in the patient with Ph-like B-ALL (left panel). The expression of S and G2 genes highlights the proliferating cells. Dot plot showing the average expression levels and cell expression proportions of *CD34* and selected cell cycle genes in the indicated clusters (right panel). The colors represent the average expression levels, and dot sizes represent the expression percentage of selected genes in the indicated clusters. **F** Trajectory analysis of B-cell clusters classified into six subtypes (n = 9,393 cells), colored by pseudotime. Solid and dotted lines represent distinct cell trajectories defined by single-cell transcriptomes. **G** Cell clusters overlaid on the tSNE representation and split by samples. **H** Violin plots show the expression of well-known B-cell marker genes. **I** A volcano plot of DEGs up-regulated (red) or down-regulated (blue) in the relapse-specific clusters (clusters 2 and 14); the top 10 up-regulated genes are labeled. The p-value is derived using the Wilcoxon rank-sum test. **J** Pathways up-regulated in the relapse-specific clusters (clusters 2 and 14). The p-value is derived using a hypergeometric test. **K** Expression levels of the relapse-specific gene *ACSM3* overlaid on the tSNE representation (left panel). Boxplot (right panel) showing significantly higher expression levels of *ACSM3* in relapsed patients with CNS-L

t-Distributed Stochastic Neighbor Embedding (tSNE) representation was divided on the basis of disease status (Fig. 2G), and all non-B cell clusters were enriched in relapse surprisingly (Additional file 1: Fig. S4A, B). Primary-enriched B cell clusters 4, 6, and 15 (> 80%), along with the low expression of *CD19* and high expression of *CD79A* and *CD10* (Fig. 2G, H), showed down-regulation in the *MYC* targets and oxidative phosphorylation pathways (Additional file 1: Fig. S4C). Differentially expressed genes (DEGs) and significantly enriched pathways for the Relapse-specific B-cell clusters 2 and 14 (> 90%), characterized by the absence of *CD20* expression, were identified (Fig. 2I, J). B-ALL patients with the high relapsed B-cell feature (Additional file 1: Additional file methods) was found to be significantly associated with a poor prognosis (*P* = 0.0077; Additional file 1: Fig. S4D). Furthermore, the expression of *ACSM3* and *HRK* were higher in relapsed B-ALL with CNS-L, indicating their potential contribution to brain infiltration in Ph-like patient (Fig. 2K, Additional file 1: Fig. S4E). Taken together, these findings uncover the single-cell transcriptome heterogeneity and dynamic evolution during disease progression.

Next, we investigated the similarities and differences between Ph-like and Ph^+^ ALL at the single-cell level [4]. A total of 25,559 cells were classified into 23 clusters which are assigned into six different cell types (Additional file 1: Fig. S5A-C). We found that Ph-like specific B-cells (cluster 1, 8) showed a higher stemness feature of comparing with other B cell clusters (Additional file 1: Fig. S5D-F). In addition, we observed that cluster 10 (CD8^+^ T cells) from diagnosis and relapse samples (Additional file 1: Fig. S5D) possessed both high NK (*KLRB1* positive) and high cytotoxicity signature scores (Additional file 1: Fig. S5G-I), consisting with those in solid tumors [10]. *CLEC2D*, as the ligand of *KLRB1* (*CD161*), is reported to be expressed in T/NK cells, germinal center (GC)-associated B cells, early plasmablasts and GC-derived lymphomas, but not in B-ALL cells [11], and protect target cells against NK cell-mediated killing as the inhibitory immune checkpoint in NK cells [12]. Our scRNA-seq data revealed that the expression level of *CLEC2D* in malignant B-ALL cells (clusters 1, 2, 3, 5, 6, 8, 13, 19 and 22), was also comparable with that of T cells (clusters 4, 9, 10 and 16) and NK cells (clusters 15 and 23) (Additional file 1: Fig. S5J), implying the potential for immune evasion.

In recent years, immune microenvironment of tumors has already become more important in cancer treatment. Previous scRNA-seq analysis has revealed the pivotal role of monocytes [4] and exhausted T cells [6] in non-Ph-like B-ALL. Our study not only demonstrated the remarkable heterogeneity of malignant B-cells, but also discovered the ectopic CD8^+^ T cells and NK cells which may contribute to immune escape. However, our work lacks a further point in time of *TPR-PDGFRB* positive Ph-like patient and more Ph-like cases for validation. Moreover, subsequent design experiments are required to better elucidate the mechanism of the abnormal *CLEC2D* expression to guide the development of relevant molecular targeting drugs.

## Conclusion

Collectively, scRNA-seq presents a comprehensive atlas and dynamic shifts of cellular composition in one Ph-like ALL patient with a novel *TPR-PDGFRB* fusion gene, which might aid in improving our understanding of the cell heterogeneity and its implications in the progression of Ph-like ALL. The integrative single-cell analysis of Ph-like and Ph^+^ ALLs revealed malignant B cells with the ectopic expression of the inhibitory receptor *CLEC2D* as a common feature, which indicating the potential role of *CLEC2D-CD161* as a novel immune checkpoint therapy target in B-ALL.

## Supplementary Information

**Additional file 1**: **Table S1.** Clinical information of the one Ph-like ALL patient at different disease stages. **Table S2.** The fusion gene list of one Ph-like ALL patient at different disease stages. **Table S3.** Summary of therapeutic regimens for the Ph-like ALL patient. **Figure.** **S1** The clinical diagnosis of one Ph-like ALL patient. (A) Diagram of the whole treatment process of the Ph-like ALL patient. NR: not remission; PR: partial remission; CR: complete molecular remission; MRD: minimal residual disease. (B) Karyotype analysis showed the normal karyotype at diagnosis for the Ph-like ALL patient. (C) Wright stain of bone marrow aspirate smear from four specimens showed the blast cells clearly. (D) Flow cytometry analysis of immunophenotypic markers for Ph-like ALL patient at diagnosis and three relapse timepoints. **Figure.** **S2** Evaluation of Ph-like expression signature and molecular marker potential for patient with novel *TPR-PDGFRB* fusion gene. (A) Correlation analysis between the *TPR-PDGFRB* positive Ph-like patient and different subtypes from TARGET-ALL-P2 cohort. Correlation coefficient was calculated between the *TPR-PDGFRB* positive Ph-like patient and each of the patients in different subtypes using person method based on the expression level of all overlapped genes. (B) Hierarchical clustering analysis between the Case 13 and other B-ALL patients for three cohorts using the genes defined previously. The red and bold line in each dendrogram for three datasets highlights the location of Case 13. (C) Sanger sequencing of the RT-PCR product validated the TPR-PDGFRB fusion junction. (D) The percentage of blasts evaluated by morphology and immuno-phenotyping at thirteen time points. (E) Amplification plot of qRT-PCR for *TPR-PDGFRB* fusion transcript and controls. qRT-PCR analysis on the standard reference sample with serial 10-fold gradient dilutions of *TPR-PDGFRB* fusion transcript copies/μl and *TPR-PDGFRB* fusion patient’s specimen at diagnosis. Additional amplifications include a no template control (NTC) and an unrelated control cDNA came from a pediatric B-ALL specimen that does not involve a *TPR-PDGFRB* fusion. (F) MRD of detected by qRT-PCR for *TPR-PDGFRB* fusion transcript. (G) MRD of detected by ddPCR for *TPR-PDGFRB* fusion transcript. **Figure.** **S3** Clusters and transcriptomic feature of different cell types in Ph-like ALL. (A) tSNE visualization of 10,273 individual cells from the patient with Ph-like ALL (*TPR-PDGFRB* fusion positive) with matched diagnosis (*n* = 4,486) and relapse (*n* = 5,787) bone marrow samples. (B) Unsupervised t-SNE plot displaying 10,273 cells from one Ph-like ALL patient at diagnosis and relapse, color-coded by 17 clusters. (C) Heatmap of the top-five genes marking 17 clusters. (D) Violin plot of selected genes which are differently expressed in classical monocytes and non-classical monocytes. (E) tSNE projections of selected T cell and NK cell genes. (F) Dot plot showing the average expression levels and cell expression proportions of selected MEP and cell cycle genes in the indicated clusters. (G) Violin plots show the expression of cluster-specific markers for memory B cells. **Figure.** **S4** The functional annotation and clinical outcomes of clusters specific for different disease status in Ph-like ALL. (A) Percentage of cell origin within each clusters (n = 17). (B) Cell types overlaid on the tSNE representation and split by samples, color-coded by cell types. For all non-B cells, the corresponding cluster numbers were labeled using the pink text and cells were indicated by the dotted oval. (C) Representative GSEA plots of three primary specific clusters (cluster 4, 6 and 15) comparing with other B cell clusters. The enrichment score (ES) and false discovery rate (FDR) are shown in the graph. (D) Survival analysis of the relapsed B-cell feature (top 10 up-regulated genes) in Figure 2I in the TARGET-ALL-P2 cohort. (E) Expression levels of relapse-specific gene *HRK* overlaid on the tSNE representation (left panel). Boxplot (right panel) showed that the higher expression of *HRK* in relapsed patients with CNS-L. **Figure.** **S5** Single-cell transcriptional profiles of Ph-like and Ph^+^ ALL patients. (A) The tSNE visualization of 25,559 individual cells from one patient with Ph-like ALL (*TPR-PDGFRB* fusion positive) and two patients with Ph^+^ ALL with matched bone marrow samples obtained at diagnosis and after relapse. (B) Unsupervised tSNE plot split into Ph-like and Ph^+^ ALL patients, displaying 25,559 cells color-coded by 23 clusters. (C) Expression levels (x-axis) of cell type-defining genes in each cluster. Violin plots showing the distribution of the normalized expression levels of genes that are color coded on the basis of the cluster, as in (B). (D) Percentage of cell origin within each clusters (n = 23). (E) A volcano plot of the DEGs that were up-regulated (red) or down-regulated (blue) in the Ph-like specific clusters (clusters 1 and 8); the top 10 up-regulated genes are labeled. The p-value is derived using the Wilcoxon rank-sum test. (F) Representative GSEA plots of Ph-like specific clusters (cluster 1, 8) comparing with other B cell clusters. The ES and FDR are shown in the graph. (G) Violin plots showing the distribution of the normalized expression levels of cytotoxic genes (left panel), NK cell genes (middle panel) and exhausted genes (right panel) in T cell (clusters 4, 9, 10, and 16) and NK cell clusters (clusters 15 and 23). (H) Scatter plot for the signature scores about cytotoxicity and NK feature in the T cell clusters. (I) tSNE visualization of cells colored by the expression of cytotoxicity or NK receptor signatures. (J) Violin plot of immune checkpoint genes including *CLEC2D* and* KLRB1*.

## Data Availability

The data and materials applied in supporting the findings in this study are available from the corresponding author upon request.

## Abbreviations

Ph-like ALL: Philadelphia chromosome-like acute lymphoblastic leukemia
cRNA-seq: Single-cell RNA sequencing
B-ALL: B-cell ALL
MRD: Minimal residue disease
TKI: Tyrosine kinase inhibitor
CR: Complete remission
CNS-L: Central nervous system leukemia
FISH: Fluorescence in-situ hybridization
RT-PCR: Reverse transcription-polymerase chain reaction
tSNE: t-Distributed Stochastic Neighbor Embedding
MEPs: Megakaryocytes-erythroid progenitors
NK: Natural killer
DEGs: Differentially expressed genes
GC: Germinal center

## References

[1] Inaba H, Pui CH (2021). Advances in the diagnosis and treatment of pediatric acute lymphoblastic leukemia. J Clin Med.

[2] Tran TH, Tasian SK (2021). Has Ph-like ALL superseded Ph+ ALL as the least favorable subtype?. Best Pract Res Clin Haematol.

[3] Slayton WB, Schultz KR, Kairalla JA, Devidas M, Mi X, Pulsipher MA (2018). Dasatinib plus intensive chemotherapy in children, adolescents, and young adults with philadelphia chromosome-positive acute lymphoblastic leukemia: results of children’s oncology group Trial AALL0622. J Clin Oncol.

[4] Witkowski MT, Dolgalev I, Evensen NA, Ma C, Chambers T, Roberts KG (2020). Extensive remodeling of the immune microenvironment in B cell acute lymphoblastic leukemia. Cancer Cell..

[5] Rabilloud T, Potier D, Pankaew S, Nozais M, Loosveld M, Payet-Bornet D (2021). Single-cell profiling identifies pre-existing CD19-negative subclones in a B-ALL patient with CD19-negative relapse after CAR-T therapy. Nat Commun.

[6] Wang X, Chen Y, Li Z, Huang B, Xu L, Lai J (2021). Single-Cell RNA-Seq of T Cells in B-ALL patients reveals an exhausted subset with remarkable heterogeneity. Adv Sci.

[7] Schwab C, Ryan SL, Chilton L, Elliott A, Murray J, Richardson S (2016). EBF1-PDGFRB fusion in pediatric B-cell precursor acute lymphoblastic leukemia (BCP-ALL): genetic profile and clinical implications. Blood.

[8] Gu Z, Churchman M, Roberts K, Li Y, Liu Y, Harvey RC (2016). Genomic analyses identify recurrent MEF2D fusions in acute lymphoblastic leukaemia. Nat Commun.

[9] Zhang J, McCastlain K, Yoshihara H, Xu B, Chang Y, Churchman ML (2016). Deregulation of DUX4 and ERG in acute lymphoblastic leukemia. Nat Genet.

[10] Mathewson ND, Ashenberg O, Tirosh I, Gritsch S, Perez EM, Marx S (2021). Inhibitory CD161 receptor identified in glioma-infiltrating T cells by single-cell analysis. Cell..

[11] Llibre A, Lopez-Macias C, Marafioti T, Mehta H, Partridge A, Kanzig C (2016). LLT1 and CD161 expression in human germinal centers promotes B cell activation and CXCR4 downregulation. J Immunol.

[12] Aldemir H, Prod'homme V, Dumaurier MJ, Retiere C, Poupon G, Cazareth J (2005). Cutting edge: lectin-like transcript 1 is a ligand for the CD161 receptor. J Immunol.

