# Supplementary material for: Single-cell heterogeneity and dynamic evolution of Ph-like acute lymphoblastic leukemia patient with novel TPR-PDGFRB fusion gene
Source: Exp Hematol Oncol. 2023 Feb 17;12:19. doi: 10.1186/s40164-023-00380-8 (PMC9936632; doi:10.1186/s40164-023-00380-8)
Supplement: Supplementary file 1 — Additional file 1: Table S1. Clinical information of the one Ph-like ALL patient at different disease stages. Table S2. The fusion gene list of one Ph-like ALL patient at different disease stages. Table S3. Summary of therapeutic regimens for the Ph-like ALL patient. Figure. S1 The clinical diagnosis of one Ph-like ALL patient. (A) Diagram of the whole treatment process of the Ph-like ALL patient. NR: not remission; PR: partial remission; CR: complete molecular remission; MRD: minimal residual disease. (B) Karyotype analysis showed the normal karyotype at diagnosis for the Ph-like ALL patient. (C) Wright stain of bone marrow aspirate smear from four specimens showed the blast cells clearly. (D) Flow cytometry analysis of immunophenotypic markers for Ph-like ALL patient at diagnosis and three relapse timepoints. Figure. S2 Evaluation of Ph-like expression signature and molecular marker potential for patient with novel TPR-PDGFRB fusion gene. (A) Correlation analysis between the TPR-PDGFRB positive Ph-like patient and different subtypes from TARGET-ALL-P2 cohort. Correlation coefficient was calculated between the TPR-PDGFRB positive Ph-like patient and each of the patients in different subtypes using person method based on the expression level of all overlapped genes. (B) Hierarchical clustering analysis between the Case 13 and other B-ALL patients for three cohorts using the genes defined previously. The red and bold line in each dendrogram for three datasets highlights the location of Case 13. (C) Sanger sequencing of the RT-PCR product validated the TPR-PDGFRB fusion junction. (D) The percentage of blasts evaluated by morphology and immuno-phenotyping at thirteen time points. (E) Amplification plot of qRT-PCR for TPR-PDGFRB fusion transcript and controls. qRT-PCR analysis on the standard reference sample with serial 10-fold gradient dilutions of TPR-PDGFRB fusion transcript copies/μl and TPR-PDGFRB fusion patient’s specimen at diagnosis. Additional amplifications i [file 40164_2023_380_MOESM1_ESM.docx]

**Additional file**

**Single-cell heterogeneity and dynamic evolution of Ph-like acute lymphoblastic leukemia patient with novel *TPR-PDGFRB* fusion gene**

Xuehong Zhang^1,2†*^, Zhijie Hou^1,2†^, Dan Huang^1^, Furong Wang^1^, Beibei Gao^1^, Chengtao Zhang^1^, Dong Zhou^1^, Jiacheng Lou^3^, Haina Wang^1^, Yuan Gao^1^, Zhijie Kang^1^, Ying Lu^1,4*^, Quentin Liu^2,5*^, Jinsong Yan^1*^

^1^ Department of Hematology, Liaoning Medical Center for Hematopoietic Stem Cell Transplantation, Dalian Key Laboratory of Hematology, Liaoning Key Laboratory of Hematopoietic Stem Cell Transplantation and Translational Medicine, Diamond Bay Institute of Hematology, the Second Hospital of Dalian Medical University, Dalian, China.

^2^ Institute of Cancer Stem Cell, Dalian Medical University, Dalian, China.

^†^Xuehong Zhang and Zhijie Hou contributed equally to the study.

^*^**Correspondence:** [zhangxuehong1984@163.com](mailto:zhangxuehong1984@163.com); [stove@shsmu.edu.cn](file:///D:\projects\合作项目\闫主任\李老师合作项目\赵京萍fusion项目\Manuscript\CCR\stove@shsmu.edu.cn); [liuq9@mail.sysu.edu.cn](mailto:liuq9@mail.sysu.edu.cn); [yanjsdmu@dmu.edu.cn](file:///C:\Users\JS%20Yan\Desktop\yanjsdmu@dmu.edu.cn).

**Lead Contact:** Jinsong Yan, [yanjsdmu@dmu.edu.cn](file:///C:\Users\JS%20Yan\Desktop\yanjsdmu@dmu.edu.cn).

**Additional file Materials and Methods**

# Contents

[Contents 2](#_Toc122011894)

[A. Supplementary Methods 3](#_Toc122011895)

[B. Supplementary Tables 8](#_Toc122011896)

[C. Supplementary Figures 14](#_Toc122011897)

[D. References 20](#_Toc122011898)

# Additional file Methods

**Patient samples**

For a *TPR-PDGFRB* positive patient, five BM samples and one cerebrospinal fluid (CSF) sample were sequentially collected corresponding to the disease status as follows: primary diagnosis (primary: 190823); complete remission (CR: 200326); relapse (1st relapse: 200201, 2nd relapse: 200414, and 3rd relapse: 200911); and central nervous system leukemia (CNS-L: 200501). The detailed treatment course and regimens are provided in Additional file 1: Table S1 and S2.

**Cytogenetics and fluorescence *in-situ* hybridization (FISH)**

R-banding karyotype analysis was conducted in the patient who was *TPR-PDGFRB* positive at diagnosis, following the standard clinical protocol, and described according to the international system for Human Cytogenetic Nomenclature. The FISH technique was used to detect the fusions using the standard clinical protocol. Briefly, 200 cells were analyzed for disruptions in *PDGFRB*. Interphase nuclei were probed using the *PDGFRB* break-apart probe (LPH031, CytoCell, Cambridge, UK) comprising a green 154-kb probe and a red 107-kb probe, which were positioned on each side of the *PDGFRB* gene.

**Sanger sequencing and MRD evaluation**

Reverse transcription-polymerase chain reaction (RT-PCR) amplification was performed using the following primers: (1) primers for the *TPR-PDGFRB* fusion transcript, forward: 5′-AGTCTGTAGGACGTGGCCTT-3′, reverse: 5′-TGGGGTCCACGTAGATGTACTC-3′; and (2) primers for *GADPH*, forward: 5′-AGCCACATCGCTCAGACAC-3′, reverse: 5′-GCCCAATACGACCAAATCC-3′. The RT-PCR products were purified using the Amicon 0.5 mL 30K Centrifugal filter (#UFC50306, Millipore), and sequencing was performed with the same primer.

Additionally, the MRD was monitored using *TPR-PDGFRB* fusion transcript copies via real-time qRT-PCR and droplet digital PCR (ddPCR). The ABI Prism 7900HT Sequence Detection System and QX200 ddPCR (Bio-Rad) were used to detect the frequency of the *TPR-PDGFRB* fusion transcripts. The primers and probes used for the *TPR-PDGFRB* fusion gene, and control ABL1 gene were as follows: 5′-CAAATACCAGTGGGAAT-3′ (*TPR-PDGFRB* forward); 5′-TCACCTTCCATCGGATCTCGTAA-3′ (*TPR-PDGFRB* reverse); 5′-FAM-TGCCAAAGCATGATGAGGATGATAAGGGAG-BHQ1-3′ (*TPR-PDGFRB* fusion); 5′-CTAAAGGTGAAAAGCTCCG-3′ (*ABL1* forward); 5′-GACTGTTGACTGGCGTGAT-3′ (*ABL1* reverse); and 5′-FAM-CCATTTTTGGTTTGGGCTTCACACCATT-TAMRA-3′ (*ABL1*-wt). The relative quantity of the qRT-PCR was calculated using the △△CT method. The ddPCR workflow was presented as described previously [[1](#_ENREF_1)], and the QuantaSoft tool (Bio-Rad) was used to quantify the ddPCR reads.

**Western blot**

The cells were harvested using lysis buffer. Subsequently, the cell lysates were subjected to sodium dodecyl sulfate-polyacrylamide gel electrophoresis (SDS-PAGE), transferred to nitrocellulose membranes, and immunoblotted with the following antibodies: *PDGFRB* (Cell Signaling Technology (CST); #3169), *TPR* (Abcam; #ab70610). The immunoblots were analyzed using the Odyssey system (LI-COR Biosciences).

**Bulk RNA library preparation and sequencing**

RNAs of the cryopreserved BMMCs were extracted using the QIAamp AllPrep RNA Mini Kit (Cat# 80204, QIAGEN), according to the manufacturer's specifications. Libraries were constructed using the TruSeq RNA Sample Preparation Kits (Illumina), and the quality of the library was assessed using Bioanalyzer 2100 (Agilent Technologies). Massively parallel RNA sequencing (RNA-seq) was performed on the NovaSeq platform (read length, paired end 150 bp; Novogene, Beijing, China).

**Bulk RNA-seq data processing**

For the gene expression, the sequencing data were mapped to the reference genome (hg38) using STAR [[2](#_ENREF_2)], and the transcript coordinates were defined on the basis of the gene annotation format file (GTF file) from GENCODE (Release 27, GRCh38). The gene abundances are presented as Reads Per Kilobase per Million mapped reads (RPKM) using the “cuffnorm” command from the Cufflinks package [[3](#_ENREF_3)]. STAR-Fusion was used to detect the fusion transcripts, the detailed results are provided in Additional file 1: Table S3.

In order to evaluate the expression similarities between *TPR-PDGFRB* positive patient and different B-ALL subtypes, the expression matirxes were downloaded from the Therapeutically Applicable Research to Generate Effective Treatments ALL Phase II (TARGET-ALL-P2: <https://ocg.cancer.gov/programs/target/>) [[4](#_ENREF_4)] and ERG dataset [[5](#_ENREF_5)] respectively. Only subtypes with specific molecular alterations and sufficient samples (*n* > 5) were retained. Finally, TARGET-ALL-P2 cohort consisted of seven subtypes including Ph^+^, Ph-like, *ETV6-RUNX1*, *TCF3-PBX1*, *ZNF384*-rearrangement, *MEF2D*-rearrangement, and *PAX5*-alteration, and the ERG dataset includes four subtypes such as Ph^+^, Ph-like, *ETV6-RUNX1*, and *ERG*-rearrangement. Person correlation coefficient was calculated between the *TPR-PDGFRB* positive patient and each of the patients in seven subtypes from TARGET-ALL-P2 cohort using the “cor” function in R (<http://cran.r-project.org/>). The patient exhibited higher correlation coefficients with Ph^+^ ALLs than with other ALL subtypes. Roberts et al. have defined the Ph-like ALL signature which consisting of 192 genes [[6](#_ENREF_6)]. Based on these 192 genes, hierarchical clustering analysis was performed between *TPR-PDGFRB* positive patient and each of the patients from two cohorts using the “hclust” function in R. This patient blended into the Ph^+^ cluster, indicating the Ph-like expression feature.

**scRNA-seq and data processing**

scRNA-seq was performed on the BMMCs from the primary and 1st relapse samples collected from a patient who was *TPR-PDGFRB* positive using the chromium system (10X Genomics). Approximately 16,000 cells from each sample were loaded onto the chip, and the library was prepared using the Chromium Single Cell 3 Reagent Kit (v3) according to the manufacturer's instructions. The library was sequenced at one full lane per sample using a HiSeq4000 platform (Illumina) with 150-bp paired-end reads. The reads were aligned to the hg38/GRCh38 reference genome, and the gene expression was quantified to generate the gene-barcode unique molecular identifier (UMI) matrices using the CellRanger software package (v3.0.1). Low-quality cells, multiple cells or doublets, and cells with > 10% of the transcripts obtained from mitochondrial genes were excluded from the subsequent analysis.

**scRNA-seq integrated analysis and unsupervised clustering**

Seurat anchor-based integration analysis was performed to eliminate the biological and technical batch effects of the samples and scRNA-seq libraries [[7](#_ENREF_7)]. A total of 10,273 cells from the primary and relapse samples obtained from the patient with Ph-like B-ALL were processed. The count data were normalized by a scale factor (10,000) followed by a natural-log transformation. The 2,000 highly variable genes (HVGs) which were detected using the “FindVariableGenes” function were subsequently used to identify the integration anchors based on the first 30 dimensions by employing the “IntegrateData” function. Principal component analysis (PCA) was performed using the “RunPCA” function to project the cells in a two-dimensional space. After a graph-based Louvain clustering, 17 clusters were partitioned using the “FindClusters” function with their 30 nearest neighbors and a resolution of 0.9. Finally, the gene expression and clustering results were visualized via t-Distributed Stochastic Neighbor Embedding (tSNE) using the RunTSNE function.

**Determination of the type and state of the cell**

To define the cell type, the results from the cell type annotation tools and the differentially expressed genes (DEGs) of clusters were taken into consideration. First, we inferred the broad cell identity of each cluster using the SingleR (v1.0.1) [[8](#_ENREF_8)] and scHCL packages (v0.1.1) [[9](#_ENREF_9)]. Then, the DEGs were identified using the Wilcoxon rank-sum test implemented using the “FindAllMarkers” function in the Seurat R package (v3.2.4); genes with a fold change (FC) >1.5 and p-value < 0.05 were included. Finally, the cell type-specific marker genes (detected in at least 5% of the cell-type cells, FC > 1.5, p-value < 0.05) were selected via pairwise comparison using the Wilcoxon method. The five genes with the highest FC were used for the heatmap exhibition utilizing the cell type average expression matrix.

A previously reported core gene set consisting of 43 G1/S genes and 54 G2/M genes [[10](#_ENREF_10)], was used to evaluate the state of the cell cycle based on the scores obtained from the Seurat “CellCycleScoring” function.

To examine the cytotoxicity, natural killer cell (NK), and inhibitory signatures within single cells, the Seurat “AddModuleScore” function was applied to score the levels of the specific signatures. The average expression levels of well-known feature genes [[11](#_ENREF_11), [12](#_ENREF_12)] including9 cytotoxic genes (*NKG7*, *GNLY*, *CST7*, *PRF1*, *GZMA*, *GZMB*, *GZMH*, *IFNG*, and *TNFSF10*), and 10 NK signature genes (*KLRD1*, *FGFBP2*, *S1PR5*, *KLRC1*, *KLRC2*, *KLRC3*, *KLRB1*, *KLRK1*, *KLRG1*, and *FCGR3A*) were used to calculate the feature scores. The cytotoxicity, NK, and inhibitory signature scores were defined on the basis of the established marker genes. A cell was considered having a high signature score if it was > 0.5.

The trajectory inference for the developmental trajectory of B cells was performed using Monocle2 (v2.14.0) [[13](#_ENREF_13)]. The “subset” function was first used to extract the normalized mRNA counts of the B cells, and an object was created according to the Monocle2 tutorial. For the pseudotime analysis, the top 1,000 HGVs were selected using the “FindVariableGenes” function to order the cells.

**Pathway analysis**

Pairwise comparison using the “wilcoxauc” function in the presto R package (v1.0.0) was performed to investigate the biological states or functional differences of the distinct cell types. The ranked gene lists ordered by using log2FC were used to investigate the MSigDB Hallmark gene sets by gene set enrichment analysis (GSEA) [[14](#_ENREF_14)]. The DEGs (FC >1.5) between the primary/relapsed specific B-cell clusters and other clusters detected using the “FindMarkers” function were enriched with the Hallmark and KEGG gene sets by using GSEA. The DEGs between the primary/relapsed specific B-cell clusters and other clusters detected via the “FindMarkers” function were enriched with the Hallmark and KEGG gene sets by using GSEA.

**Survival analysis**

Survival analysis was performed on the bulk RNA cohort TARGET-ALL-P2. The gene expression and survival data were downloaded using the R function “GDCdownload” in the TCGAbiolinks package (v2.18.0). The relapsed B-cell feature was defined as the mean log2 (RPKM + 1) normalized expression of the folllowing DEGs (*ACSM3*, *HRK*, *IGLC3*, *DPEP1*, *NSMCE1*, *IGLC2*, *IGKC*, *CD9*, *DDX54*, and *BTG2*) between the relapsed specific clusters and other clusters. The samples were divided into high and low expression groups based on their median values. A Kaplan-Meier survival curve with the events table showing differences in survival time was plotted using the survminer package (v0.4.9). The survival risk and statistical significance were determined according to the hazard ratio (HR) and log-rank p-values reported in the survival package in R (v3.2-11).

**Integrated analysis of Matthew's dataset**

In order to compare the cell identity between the patients with Ph-like and Ph^+^ B-ALL, Matthew's dataset GSE130116 was downloaded from the Gene Expression Omnibus and re-analyzed. Then, the scRNA-seq data of the BM samples from two patients with Ph^+^ B-ALL at diagnosis and relapse were included for subsequent analyses. The criteria for quality control were as follows: doublets or multiple cells, cells with < 200 genes, and cells with >10% of reads mapped to mitochondrial genes. Genes that were detected in fewer than 60 cells were filtered out. For the integrated analysis, the Seurat3 “anchor” method was used to remove the batch effect. The integrated dataset was processed for dimension reduction and cell clustering at a resolution of 0.7. Finally, cell type annotation and cell state evaluations, including the cell cycle score, signature score, and developmental trajectory, were performed as described above.

# Additional file Tables

## Table S1. Clinical information of one Ph-like ALL patient at different disease stages.

| Date | | Status | Morphology blast of BM (%) | | | | | WBC  (*10^9^/L) | RBC  (*10^12^/L) | Hb  (g/L) | PLT  (*10^9^/L) | Immunophenotype blast (%) | Cytogenetics | *TPR-PDGFRB* |
| --- | --- | --- | --- | --- | --- | --- | --- | --- | --- | --- | --- | --- | --- | --- |
|  |  |  |  | | | | |  |  |  |  |  |  |  |
|  |  |  | lymphoblast (%) | | Naive lymphocyte (%) | | Mature lymphocyte (%) |  |  |  |  |  |  |  |
| 19.08.23 | | Primary | 74 | |  | | 25.5 | 563.45 | 1.53 | 42 | 34 | 91.38 | 46, XX[20] | + |
| 19.09.18 | | CR | 0 | | 0 | | 8 | 77.5 | 2.67 | 81 | 22 | 0 | - | - |
| 19.10.18 | | CR | 0 | | 0 | | 12.5 | 6.57 | 2.73 | 84 | 234 | 0 | - | - |
| 19.11.08 | | CR | 0 | | 1.5 | | 19.5 | 2.86 | 2.74 | 88 | 221 | 2.31 | - | - |
| 19.12.10 | | CR | 0 | | 1 | | 10 | 9.36 | 2.75 | 90 | 310 | 0 | - | - |
| 20.02.01 | | Relapse 1st | 33.5 | | 5 | | 4 | 21.99 | 2.47 | 80 | 338 | 49 | - | + |
| 20.02.25 | | CR | 0 | | 2 | | 98 | 0.12 | 2.9 | 91 | 46 | 0 | - | - |
| 20.03.26 | | CR | 0 | | 0.5 | | 8.5 | 1.64 | 1.53 | 45 | 40 | 0 | - | - |
| 20.04.14 | | Relapse 2nd | 38 | | 0 | | 3.5 | 3.21 | 2.19 | 70 | 137 | 10.92 | - | + |
| 20.04.22 | | NR | 25.5 | | 16 | | 24 | 2.99 | 2.21 | 72 | 150 | 14.9 | - | + |
| 20.04.28 | | NR | 13.5 | | 12.5 | | 27.5 | 2.68 | 2.25 | 78 | 161 | 9.77 | - | + |
| 20.05.01 | | CNS-L |  | |  | |  | 2.45 | 2.25 | 78 | 153 | 96.55 (CSF) | - | + |
| 20.05.07 | | CNS-L |  | |  | |  | 0.24 | 2.14 | 78 | 74 | 19.3 (CSF) | - | + |
| 20.05.20 | | CR | 0 | | 0.5 | | 16.5 | 2.58 | 2.34 | 73 | 39 |  | - | - |
| 20.06.04 | | CR | 0 | | 0 | | 23.5 | 2.66 | 1.26 | 38 | 55 |  | - | - |
| 20.06.18 | CR | | 0 | 0 | | 6.5 | | 6.46 | 1.78 | 53 | 54 |  | - | - |
| 20.07.07 | CR | | 0 | 0 | | 18.5 | | 8.53 | 2.96 | 90 | 41 | 5.14 | - | - |
| 20.08.05 | CR | | 0 | 0.5 | | 16.5 | | 2.58 | 2.34 | 73 | 39 |  | - | - |
| 20.09.11 | Relapse 3rd | | 78.5 | 3 | | 6.5 | | 2.66 | 1.26 | 38 | 55 | 74.2 | - | + |
| 20.09.28 | NR | |  | 0 | | 6.5 | | 6.46 | 1.78 | 53 | 54 |  | - | + |

## Table S2. The fusion gene list of one Ph-like ALL patient at different disease stages.

| **Sample** | **FusionName** | **JunctionReadCount** | **SpanningFragCount** | **LeftGene** | **LeftBreakpoint** | **RightGene** | **RightBreakpoint** | **annots** |
| --- | --- | --- | --- | --- | --- | --- | --- | --- |
| Primary_190823 | TRDV2-TRAC | 198 | 69 | TRDV2 | chr14:22422881:+ | TRAC | chr14:22547506:+ | INFRAME |
| Primary_190823 | TPR-PDGFRB | 66 | 13 | TPR | chr1:186320312:- | PDGFRB | chr5:150126614:- | INFRAME |
| Primary_190823 | AC019118.2-LINC01250 | 19 | 4 | AC019118.2 | chr2:3145547:- | LINC01250 | chr2:2966675:- | . |
| Primary_190823 | HSDL2-KIAA1958 | 8 | 0 | HSDL2 | chr9:112380180:+ | KIAA1958 | chr9:112645650:+ | FRAMESHIFT |
| Primary_190823 | MTAP-CDKN2B-AS1 | 3 | 2 | MTAP | chr9:21859425:+ | CDKN2B-AS1 | chr9:22023944:+ | . |
| Relapse.1st_200201 | TRDV2-TRAC | 241 | 21 | TRDV2 | chr14:22422881:+ | TRAC | chr14:22547506:+ | INFRAME |
| Relapse.1st_200201 | TPR-PDGFRB | 38 | 5 | TPR | chr1:186320312:- | PDGFRB | chr5:150126614:- | INFRAME |
| Relapse.1st_200201 | AC019118.2-LINC01250 | 28 | 5 | AC019118.2 | chr2:3145547:- | LINC01250 | chr2:2966675:- | . |
| Relapse.1st_200201 | HSDL2-KIAA1958 | 11 | 0 | HSDL2 | chr9:112380180:+ | KIAA1958 | chr9:112645650:+ | FRAMESHIFT |
| Relapse.2nd_200414 | TRDV2-TRAC | 57 | 4 | TRDV2 | chr14:22422881:+ | TRAC | chr14:22547506:+ | INFRAME |
| Relapse.2nd_200414 | AC019118.2-LINC01250 | 5 | 2 | AC019118.2 | chr2:3145547:- | LINC01250 | chr2:2966675:- | . |
| Relapse.2nd_200414 | TPR-PDGFRB | 4 | 1 | TPR | chr1:186320312:- | PDGFRB | chr5:150126614:- | INFRAME |
| Relapse.2nd_200414 | CHD3-CIC | 2 | 3 | CHD3 | chr17:7885083:+ | CIC | chr19:42286771:+ | INFRAME |
| Relapse.2nd_200414 | CFL1-ZFAND5 | 4 | 0 | CFL1 | chr11:65858097:- | ZFAND5 | chr9:72360787:- | INFRAME |
| Relapse.2nd_200414 | DTX3L-GTF2E1 | 4 | 0 | DTX3L | chr3:122564613:+ | GTF2E1 | chr3:120750523:+ | . |
| Relapse.2nd_200414 | TCF25-PTMA | 4 | 0 | TCF25 | chr16:89873859:+ | PTMA | chr2:231711348:+ | INFRAME |
| Relapse.2nd_200414 | KMT2E-TRRAP | 4 | 0 | KMT2E | chr7:105014535:+ | TRRAP | chr7:99031345:+ | . |
| CSF_200501 | TRDV2-TRAC | 991 | 41 | TRDV2 | chr14:22422881:+ | TRAC | chr14:22547506:+ | INFRAME |
| CSF_200501 | TPR-PDGFRB | 30 | 9 | TPR | chr1:186320312:- | PDGFRB | chr5:150126614:- | INFRAME |
| CSF_200501 | AP003086.1-GAB2 | 14 | 1 | AP003086.1 | chr11:78425337:- | GAB2 | chr11:78280901:- | . |
| CSF_200501 | AC019118.2-LINC01250 | 8 | 1 | AC019118.2 | chr2:3145547:- | LINC01250 | chr2:2966675:- | . |
| CSF_200501 | HSDL2-KIAA1958 | 9 | 0 | HSDL2 | chr9:112380180:+ | KIAA1958 | chr9:112645650:+ | FRAMESHIFT |
| CSF_200501 | PDGFRB-TPR | 9 | 0 | PDGFRB | chr5:150129863:- | TPR | chr1:186318828:- | FRAMESHIFT |
| CSF_200501 | NOP53-DHX34 | 4 | 2 | NOP53 | chr19:47745783:+ | DHX34 | chr19:47381980:+ | FRAMESHIFT |
| CSF_200501 | MTAP-CDKN2B-AS1 | 4 | 0 | MTAP | chr9:21859425:+ | CDKN2B-AS1 | chr9:22029433:+ | . |
| Relapse.3rd_200911 | TRDV2-TRAC | 210 | 7 | TRDV2 | chr14:22422881:+ | TRAC | chr14:22547506:+ | INFRAME |
| Relapse.3rd_200911 | TPR-PDGFRB | 36 | 14 | TPR | chr1:186320312:- | PDGFRB | chr5:150126614:- | INFRAME |
| Relapse.3rd_200911 | HSDL2-KIAA1958 | 12 | 0 | HSDL2 | chr9:112380180:+ | KIAA1958 | chr9:112645650:+ | FRAMESHIFT |
| Relapse.3rd_200911 | ZBTB40-MYRIP | 10 | 0 | ZBTB40 | chr1:22452004:+ | MYRIP | chr3:40044050:+ | . |
| Relapse.3rd_200911 | KLF10-YWHAZ | 8 | 0 | KLF10 | chr8:102655566:- | YWHAZ | chr8:100948900:- | . |
| Relapse.3rd_200911 | MBP-CTDP1 | 5 | 0 | MBP | chr18:77132580:- | CTDP1 | chr18:79695225:+ | . |
| Relapse.3rd_200911 | YPEL5-KLF11 | 4 | 0 | YPEL5 | chr2:30147062:+ | KLF11 | chr2:10046150:+ | . |
| Relapse.3rd_200911 | GABPB1-B2M | 4 | 0 | GABPB1 | chr15:50354985:- | B2M | chr15:44715423:+ | . |
| Relapse.3rd_200911 | OAZ1-LIMD2 | 4 | 0 | OAZ1 | chr19:2269744:+ | LIMD2 | chr17:63699348:- | . |
| Relapse.3rd_200911 | AC019118.2-LINC01250 | 4 | 0 | AC019118.2 | chr2:3145547:- | LINC01250 | chr2:2966675:- | . |
| Relapse.3rd_200911 | S100A6-TSC22D3 | 4 | 0 | S100A6 | chr1:153534638:- | TSC22D3 | chrX:107715950:- | . |
| Relapse.3rd_200911 | ST3GAL1-PTMAP2 | 4 | 0 | ST3GAL1 | chr8:133571693:- | PTMAP2 | chr5:118973841:+ | . |

## Table S3. Summary of therapeutic regimens for the Ph-like patient.

| **Therapeutic regimen** | **Therapeutic regimen** | **Therapeutic start date** | **Usage** |
| --- | --- | --- | --- |
| hyperCVAD | C: Cyclophosphamide,300mg/m^2^,0.45g,q12h,d1-d3 | 2019.8.29 | ivgtt |
|  | V: Vincristine,1.5mg/m^2^,2mg,qd,d4,d11 |  | iv |
|  | P: Pirarubicin,50mg/m^2^,80mg,qd,d4 |  | ivgtt |
|  | D: Dexamethasone,0.2mg/kg,40mg,qd,d1-4,d11-14 |  | ivgtt |
| COP | C: Cyclophosphamide,750mg/m^2^,1.1g,qd,d1 | 2019.10.24 | ivgtt |
|  | V: Vincristine,1.4mg/m^2^,2mg,qd,d1 |  | iv |
|  | D: Dexamethasone,0.2mg/kg,10mg,qd,d1-3 |  | ivgtt |
| hyperCVAD | C: Cyclophosphamide,300mg/m^2^,0.45g,q12h,d1-d3 | 2019.11.13 | ivgtt |
|  | V: Vincristine,1.5mg/m^2^,2mg,qd,d4,d11 |  | iv |
|  | P: Pirarubicin,50mg/m^2^,80mg,qd,d4 |  | ivgtt |
|  | D: Dexamethasone,0.2mg/kg,40mg,qd,d1-4,d11-14 |  | ivgtt |
| VP+FLAG | V: Vincristine,1.4mg/m^2^,2mg,qd,d-5 | 2019.12.17 | ivgtt |
|  | D: Dexamethasone,0.2mg/kg,10mg,qd,d-5~-1 |  | iv |
|  | F: Fludarabine, 50mg/m^2^,qd, d1-d5 |  | ivgtt |
|  | C: Cytarabine,1mg/m^2^,1.5mg,qd,d1-d5 |  | ivgtt |
|  | G: G-CSF, 5ug/kg,300ug/d, d1-d5 |  | ih |
| VDLP | V: Vincristine,1.5mg/m^2^,2mg,qd,d1,d8,d15,d22 | 2020.2.04 | ivgtt |
|  | P: Pirarubicin,60mg/m^2^,80mg,qd,d1,d8,d15,d22 |  | iv |
|  | D: Dexamethasone,0.2mg/kg,10mg,qd,d1-28 |  | ivgtt |
|  | L: Pegaspargase,5ml/3750IU,qd,d17 |  | ivgtt |
| TKI | Imatinib, 400mg, qd | 2020.4.15-2020.4.30 |  |
| DOCPL | V: Vincristine,1.5mg/m^2^,2mg,qd,d1,d8,d15,d22 | 2020.5.02 | iv |
|  | P: Pirarubicin,30mg/m^2^,45mg,qd,d1,d3,d15,d17 |  | ivgtt |
|  | D: Dexamethasone,0.2mg/kg,10mg,qd,d1-14 |  | ivgtt |
|  | L: Pegaspargase,5ml/3750IU,qd,d4,d15 |  | ih |
|  | C: Cyclophosphamide,0.75g/m^2^,1.1g,qd,d1,d15 |  | ivgtt |
| TKI | Dasatinib, 50mg, q12h | 2020.05.06 |  |
| no treatment |  | 2020.08.15 |  |
| TKI | Ponatinib, 45 mg, qd | 2020.09.15 |  |
| no treatment |  | 2020.09.30 |  |
| Dead |  | 2020.10.20 |  |

# C. Additional file Figures


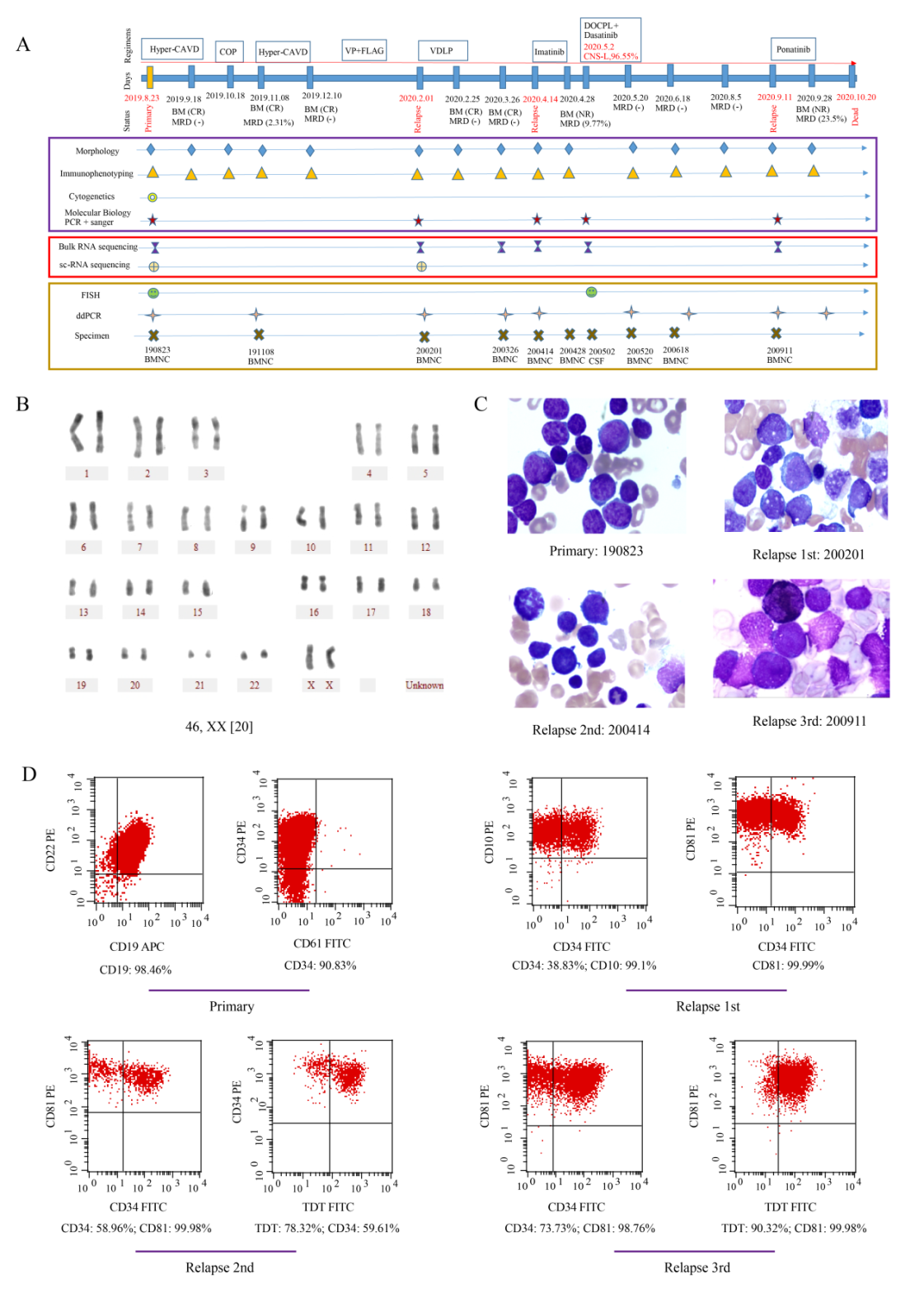


**Figure S1. The clinical information of a Ph-like ALL patient.**

1. Diagram of the whole treatment process of the Ph-like ALL patient. NR: not remission; PR: partial remission; CR: complete molecular remission; MRD: minimal residual disease.
2. Karyotype analysis showed the normal karyotype at diagnosis for the Ph-like ALL patient.
3. Wright stain of bone marrow aspirate smear from four specimens showed the blast cells clearly.
4. Flow cytometry analysis of immunophenotypic markers for Ph-like ALL patient at diagnosis and three relapse timepoints.


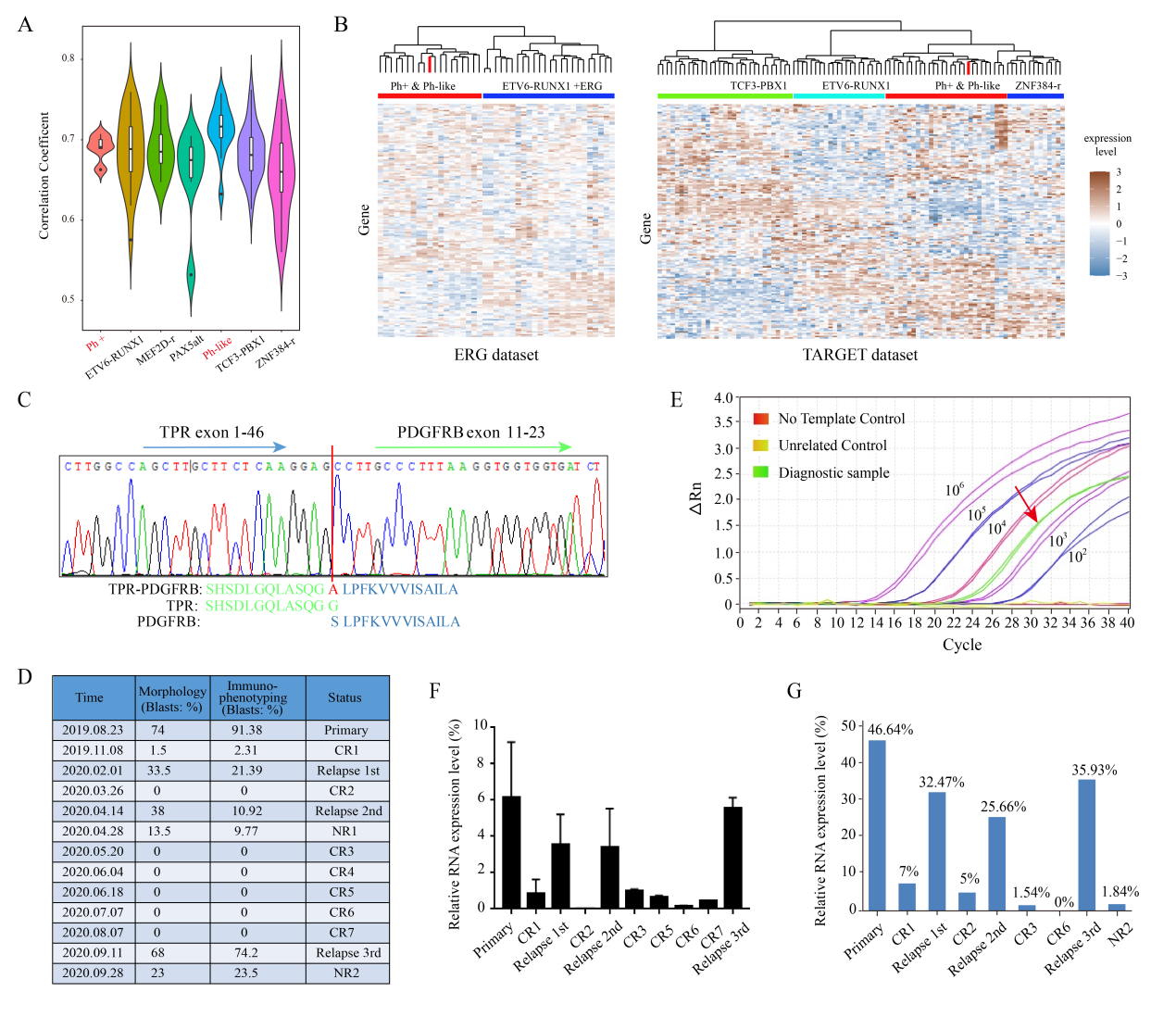


**Figure S2. Evaluation of Ph-like expression signature and molecular marker potential for patient with novel *TPR-PDGFRB* fusion gene.**

1. Correlation analysis between the *TPR-PDGFRB* positive Ph-like patient and different subtypes from TARGET-ALL-P2 cohort. Correlation coefficient was calculated between the *TPR-PDGFRB* positive Ph-like patient and each of the patients in different subtypes using person method based on the expression level of all overlapped genes.
2. Hierarchical clustering analysis between the *TPR-PDGFRB* positive Ph-like patient and other B-ALL patients for two cohorts using the genes defined previously. The red and bold line in each dendrogram for three datasets highlights the location of *TPR-PDGFRB* positive Ph-like patient.
3. Sanger sequencing of the RT-PCR product validated the *TPR-PDGFRB* fusion junction.
4. The percentage of blasts evaluated by morphology and immuno-phenotyping at thirteen time points. NR, not remission; PR: partial remission; CR: complete molecular remission.
5. Amplification plot of qRT-PCR for *TPR-PDGFRB* fusion transcript and controls. qRT-PCR analysis on the standard reference sample with serial 10-fold gradient dilutions of *TPR-PDGFRB* fusion transcript copies and *TPR-PDGFRB* fusion patient’s specimen at diagnosis (red arrow). Additional amplifications include a no template control (NTC) and an unrelated control cDNA came from a pediatric B-ALL specimen that does not involve a *TPR-PDGFRB* fusion.
6. MRD of detected by qRT-PCR for *TPR-PDGFRB* fusion transcript.
7. MRD of detected by ddPCR for *TPR-PDGFRB* fusion transcript.


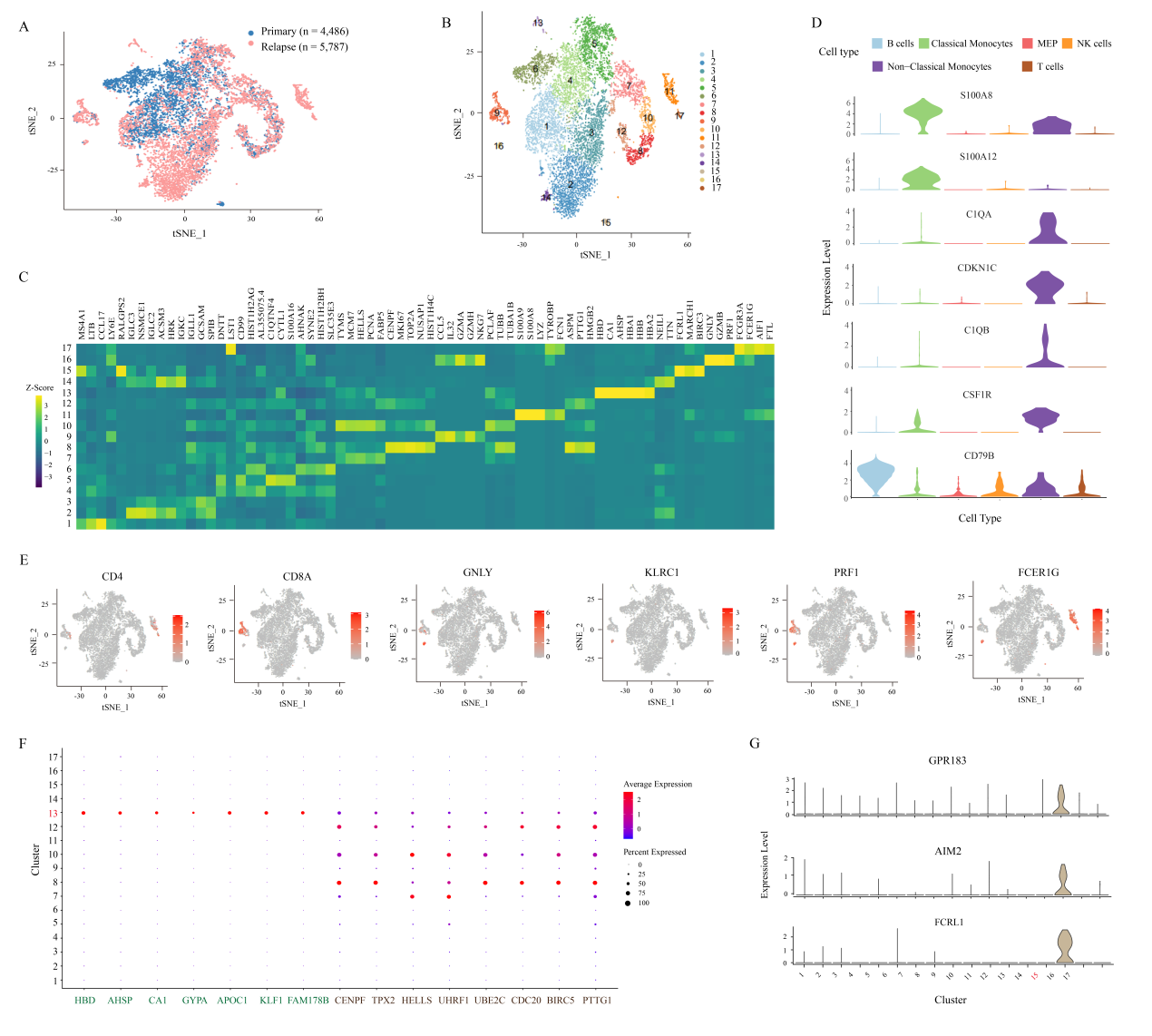


**Figure S3. Clusters and transcriptomic feature of different cell types in Ph-like ALL.**

1. tSNE visualization of 10,273 individual cells from the patient with Ph-like ALL (*TPR-PDGFRB* fusion positive) with matched diagnosis (*n* = 4,486) and relapse (*n* = 5,787) bone marrow samples.
2. Unsupervised t-SNE plot displaying 10,273 cells from Ph-like ALL patient at diagnosis and relapse, color-coded by 17 clusters.
3. Heatmap of the top-five genes marking 17 clusters.
4. Violin plot of selected genes which are differently expressed in classical monocytes and non-classical monocytes.
5. tSNE projections of selected T cell and NK cell genes.
6. Dot plot showing the average expression levels and cell expression proportions of selected MEP and cell cycle genes in the indicated clusters.
7. Violin plots show the expression of cluster-specific markers for memory B cells.

**
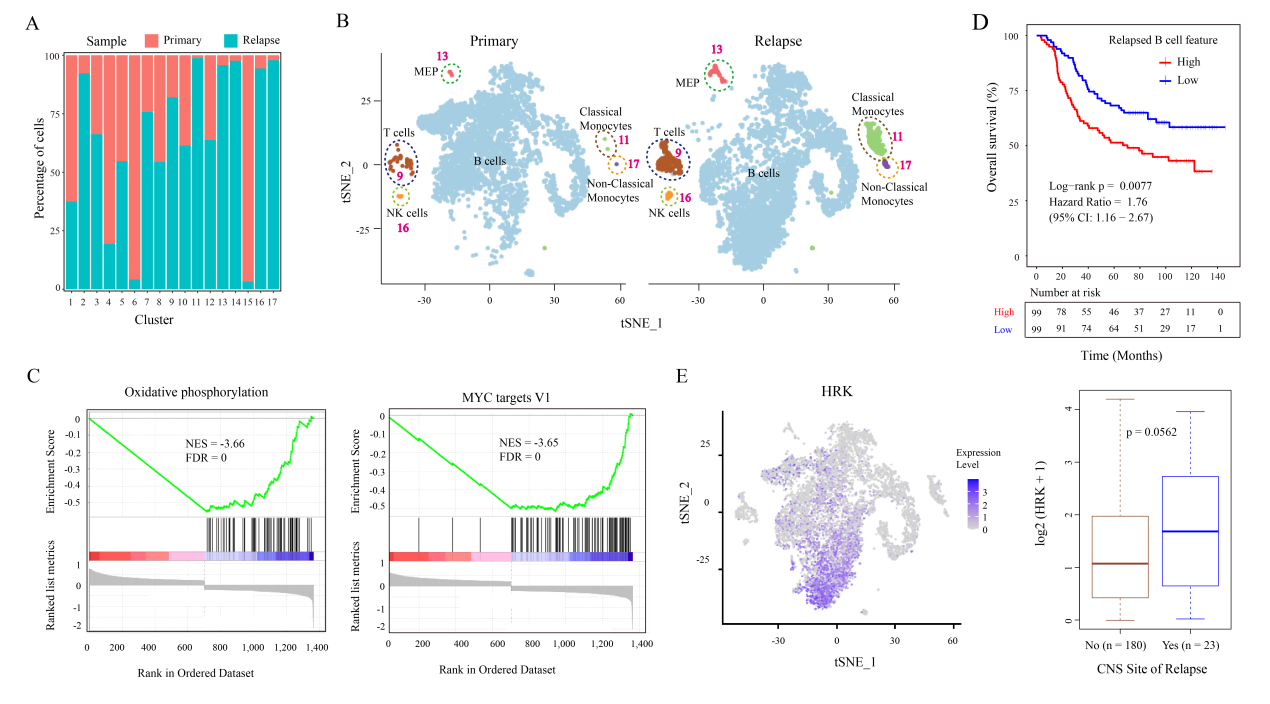
**

**Figure S4. The functional annotation and clinical outcomes of clusters specific for different disease status in Ph-like ALL.**

1. Percentage of cell origin within each clusters (*n* = 17).
2. Cell types overlaid on the tSNE representation and split by samples, color-coded by cell types. For all non-B cells, the corresponding cluster numbers were labeled using the pink text and cells were indicated by the dotted oval.
3. Representative GSEA plots of three primary specific clusters (cluster 4, 6 and 15) comparing with other B cell clusters. The enrichment score (ES) and false discovery rate (FDR) are shown in the graph.
4. Survival analysis of the relapsed B-cell feature (top 10 up-regulated genes) in **Figure 2I** in the TARGET-ALL-P2 cohort.
5. Expression levels of relapse-specific gene *HRK* overlaid on the tSNE representation (left panel). Boxplot (right panel) showed that the higher expression of *HRK* in relapsed patients with CNS-L.


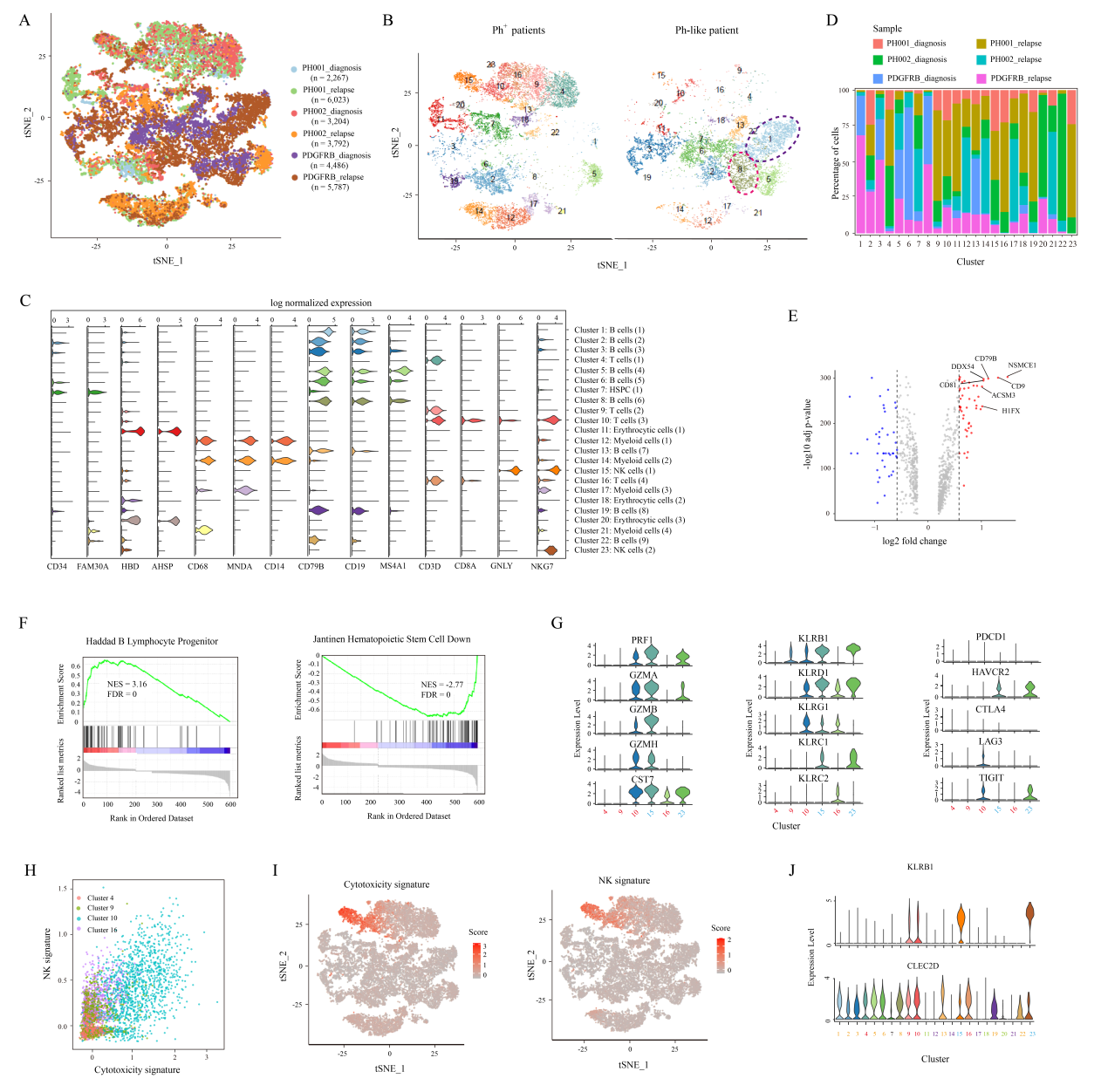


**Figure S5. Single-cell transcriptional profiles of Ph-like and Ph^+^ ALL patients.**

1. The tSNE visualization of 25,559 individual cells from one patient with Ph-like ALL (TPR-PDGFRB fusion positive) and two patients with Ph^+^ ALL with matched bone marrow samples obtained at diagnosis and after relapse.
2. Unsupervised tSNE plot split into Ph-like and Ph^+^ ALL patients, displaying 25,559 cells color-coded by 23 clusters.
3. Expression levels (x-axis) of cell type-defining genes in each cluster. Violin plots showing the distribution of the normalized expression levels of genes that are color coded on the basis of the cluster, as in (B).
4. Percentage of cell origin within each clusters (*n* = 23).
5. A volcano plot of the DEGs that were up-regulated (red) or down-regulated (blue) in the Ph-like specific clusters (clusters 1 and 8); the top 10 up-regulated genes are labeled. The p-value is derived using the Wilcoxon rank-sum test.
6. Representative GSEA plots of Ph-like specific clusters (cluster 1, 8) comparing with other B cell clusters. The enrichment score (ES) and nominal p-values are shown in the graph.
7. Violin plots showing the distribution of the normalized expression levels of cytotoxic genes (left panel), NK cell genes (middle panel) and exhausted genes (right panel) in T cell (clusters 4, 9, 10, and 16) and NK cell clusters (clusters 15 and 23).
8. Scatter plot for the signature scores about cytotoxicity and NK feature in the T cell clusters.
9. tSNE visualization of cells colored by the expression of cytotoxicity or NK receptor signatures.
10. Violin plot of immune checkpoint genes including *CLEC2D*, and *KLRB1*.

# D. References

1. Hindson BJ, Ness KD, Masquelier DA, Belgrader P, Heredia NJ, Makarewicz AJ, et al. High-throughput droplet digital PCR system for absolute quantitation of DNA copy number. Anal Chem. 2011;83(22):8604-10.

2. Dobin A, Davis CA, Schlesinger F, Drenkow J, Zaleski C, Jha S, et al. STAR: ultrafast universal RNA-seq aligner. Bioinformatics. 2013;29(1):15-21.

3. Trapnell C, Williams BA, Pertea G, Mortazavi A, Kwan G, van Baren MJ, et al. Transcript assembly and quantification by RNA-Seq reveals unannotated transcripts and isoform switching during cell differentiation. Nat Biotechnol. 2010;28(5):511-5.

4. Gu Z, Churchman M, Roberts K, Li Y, Liu Y, Harvey RC, et al. Genomic analyses identify recurrent MEF2D fusions in acute lymphoblastic leukaemia. Nat Commun. 2016;7:13331.

5. Zhang J, McCastlain K, Yoshihara H, Xu B, Chang Y, Churchman ML, et al. Deregulation of DUX4 and ERG in acute lymphoblastic leukemia. Nat Genet. 2016;48(12):1481-1489.

6. Roberts KG, Morin RD, Zhang J, Hirst M, Zhao Y, Su X, et al. Genetic alterations activating kinase and cytokine receptor signaling in high-risk acute lymphoblastic leukemia. Cancer Cell. 2012;22(2):153-66.

7. Stuart T, Butler A, Hoffman P, Hafemeister C, Papalexi E, Mauck WM, 3rd, et al. Comprehensive Integration of Single-Cell Data. Cell. 2019;177(7):1888-1902 e21.

8. Aran D, Looney AP, Liu L, Wu E, Fong V, Hsu A, et al. Reference-based analysis of lung single-cell sequencing reveals a transitional profibrotic macrophage. Nat Immunol. 2019;20(2):163-172.

9. Han X, Zhou Z, Fei L, Sun H, Wang R, Chen Y, et al. Construction of a human cell landscape at single-cell level. Nature. 2020;581(7808):303-309.

10. Tirosh I, Izar B, Prakadan SM, Wadsworth MH, 2nd, Treacy D, Trombetta JJ, et al. Dissecting the multicellular ecosystem of metastatic melanoma by single-cell RNA-seq. Science. 2016;352(6282):189-96.

11. Sun Y, Wu L, Zhong Y, Zhou K, Hou Y, Wang Z, et al. Single-cell landscape of the ecosystem in early-relapse hepatocellular carcinoma. Cell. 2021;184(2):404-421 e16.

12. Mathewson ND, Ashenberg O, Tirosh I, Gritsch S, Perez EM, Marx S, et al. Inhibitory CD161 receptor identified in glioma-infiltrating T cells by single-cell analysis. Cell. 2021;184(5):1281-1298 e26.

13. Qiu X, Mao Q, Tang Y, Wang L, Chawla R, Pliner HA, et al. Reversed graph embedding resolves complex single-cell trajectories. Nat Methods. 2017;14(10):979-982.

14. Subramanian A, Tamayo P, Mootha VK, Mukherjee S, Ebert BL, Gillette MA, et al. Gene set enrichment analysis: a knowledge-based approach for interpreting genome-wide expression profiles. Proc Natl Acad Sci U S A. 2005;102(43):15545-50.
